# Combination Therapy of Novel Oncolytic Adenovirus with Anti-PD1 Resulted in Enhanced Anti-Cancer Effect in Syngeneic Immunocompetent Melanoma Mouse Model

**DOI:** 10.3390/pharmaceutics13040547

**Published:** 2021-04-14

**Authors:** Mariangela Garofalo, Laura Bertinato, Monika Staniszewska, Magdalena Wieczorek, Stefano Salmaso, Silke Schrom, Beate Rinner, Katarzyna Wanda Pancer, Lukasz Kuryk

**Affiliations:** 1 Department of Pharmaceutical and Pharmacological Sciences, University of Padova, Via F. Marzolo 5, 35131 Padova, Italy; laura.bertinato@studenti.unipd.it (L.B.); stefano.salmaso@unipd.it (S.S.); 2 Centre for Advanced Materials and Technologies, Warsaw University of Technology, Poleczki 19, 02-822 Warsaw, Poland; monika.staniszewska@pw.edu.pl; 3 Department of Virology, National Institute of Public Health—National Institute of Hygiene, Chocimska 24, 00-791 Warsaw, Poland; mrechnio@pzh.gov.pl (M.W.); kpancer@pzh.gov.pl (K.W.P.); 4 Division of Biomedical Research, Medical University of Graz, Roseggerweg 48, 8036 Graz, Austria; silke.schrom@medunigraz.at (S.S.); beate.rinner@medunigraz.at (B.R.); 5 Clinical Science, Targovax Oy, Lars Sonckin kaari 14, 02600 Espoo, Finland

**Keywords:** melanoma, immunotherapy, oncolytic adenovirus, PD-1 inhibitors, combinatory therapy, ICOSL, CD40L

## Abstract

Malignant melanoma, an aggressive form of skin cancer, has a low five-year survival rate in patients with advanced disease. Immunotherapy represents a promising approach to improve survival rates among patients at advanced stage. Herein, the aim of the study was to design and produce, by using engineering tools, a novel oncolytic adenovirus AdV-D24- inducible co-stimulator ligand (ICOSL)-CD40L expressing potent co-stimulatory molecules enhancing clinical efficacy through the modulation of anti-cancer immune responses. Firstly, we demonstrated the vector’s identity and genetic stability by restriction enzyme assay and sequencing, then, by performing in vitro and in vivo pre-clinical studies we explored the anti-cancer efficacy of the virus alone or in combination with anti PD-1 inhibitor in human melanoma cell lines, i.e., MUG Mel-1 and MUG Mel-2, and in immunocompetent C57BL/6 melanoma B16V mouse model. We showed that both monotherapy and combination approaches exhibit enhanced anti-cancer ability and immunogenic cell death in in vitro settings. Furthermore, AdV-D24-ICOSL-CD40L combined with anti PD-1 revealed a fall in tumor volume and 100% survival in in vivo context, thus suggesting enhanced efficacy and survival via complementary anti-cancer properties of those agents in melanoma therapy. Collectively, the novel oncolytic vector AdV-D24-ICOSL-CD40L alone or in combination with anticancer drugs, such as check point inhibitors, may open novel therapeutic perspectives for the treatment of melanoma.

## 1. Introduction

Malignant melanoma is considered to be the most dangerous type of skin cancer that develops from the melanocytes, the pigment-producing cells [1,2,3]. In most cases, melanoma occurs in the skin [2], however, it can also appear in the mouth, intestines, and in the eye (known as uveal melanoma) [1,2]. Around the world, as of 2012, about 232,000 people were newly diagnosed with melanoma. The highest numbers of melanoma cases occur in New Zealand (the overall annual age-standardized rate (ASR) is 35.8), Australia (ASR = 34.9) [4,5,6], the northern part of Europe (ASR = 14.6) and in North America (ASR = 13.8); however, in Asia (ASR = 1.7), Africa (ASR = 1.1), and South America (ASR = 2.5) cases are comparatively less [4,5]. Additionally, men are more commonly affected by this disease compared to women (1.6 times) [1,2,6].

The rise of immune checkpoint inhibitors (ICIs) has significantly improved the treatment paradigm for melanoma [7]. The recent advances have been achieved due to adoption of antibodies against the immune checkpoints: programmed cell death protein-1 (anti-PD-1, pembrolizumab, nivolumab), cytotoxic T-lymphocyte-associated protein-4 (anti-CTLA-4, ipilimumab) and programmed death-ligand 1 (anti-PD-L1, atezolizumab, durvalumab, avelumab) [7,8]. These ICIs have shown profound clinical efficacy in metastatic melanoma by reversing effector T-cell exhaustion and dysfunction, leading to the improvement of their anti-tumoral properties, thereby increasing T-cell activation [9]. However, unfortunately, from 40%–60% of melanoma patients do not gain any significant therapeutic benefit and a profound proportion of responders observe tumor relapse within 2 years [9,10,11]. The response rates to ICI monotherapy are low, in part due to the presence of a “cold” immune tumor microenvironment (TME) [12]. Consequently, novel and more efficient therapeutics strategies and synergistic combination therapies are in high demand [9,12].

The fast-accelerating oncolytic virotherapy is a promising anti-cancer strategy. Oncolytic viruses are naturally occurring or genetically modified viruses that infect, replicate in, and kill cancer cells without harming normal cells [13,14,15]. Significant advances in gene engineering capabilities and refinements in vector production have been made within the last few decades [16,17,18], especially because of a better understanding of how oncolytic viruses modulate the TME, which has raised the field “oncolytic immunotherapy”. Oncolytic adenoviruses after their injection to the tumor lesion lead to increased levels of proinflammatory cytokines and an influx of NK cells, T cells, and antigen-presenting cells (APC) [7,19]. Notably, PD-L1 expression is known to elevate on cancer cells and immune cells following viral infection [20,21]. Taken together, these cascades or reactions modulate the TME and reverse it from “cold” to “hot” with a development of cytokines and immune effectors. Talimogene laherparepvec (T-VEC; Imlygic™), is a genetically engineered herpes simplex virus, type 1, and is the first oncolytic virus therapy to be approved for the treatment of advanced melanoma by the US FDA. T-VEC showed improvement in durable response rate (DRR), objective response rate (ORR), and progression-free survival (PFS) in a randomized phase III clinical trial for patients with advanced melanoma [22].

It has been shown that ICIs work better in the presence of lymphocytic infiltrate, which is not always the case in many cancer types, including melanoma [20,23]. Oncolytic viruses (OVs) induce anti-cancer immune responses that enhance the efficacy of checkpoint inhibitors. For this reason, the combinatory therapy of oncolytic vectors with ICI is considered to be a promising regime in melanoma therapy. Furthermore, the efficacy of combining OVs and ICIs has been shown in pre-clinical findings, and there are currently many ongoing clinical trials testing combination therapies with promising results with ICIs [24,25].

Nevertheless, despite extensive research, oncolytic virotherapy has shown limited efficacy against solid tumors as monotherapy [26,27]. Therefore, the development of novel and more potent oncolytic vectors is highly needed. Additionally, combinatory therapies are considered to be the future direction in cancer treatment. In fact, T-VEC demonstrated improved survival outcomes for unresectable melanoma in combination [28].

In the present preclinical study, we engineered a novel oncolytic vector, expressing two potent co-stimulatory ligands aiming at enhancing anti-cancer immune responses by T cell co-activation. Therefore, in this study we investigated whether the targeted delivery of co-stimulatory proteins mediated by OVs may improve anti-cancer immune responses to the melanomas and reduce any potential undesired off-target effects. To this aim, the novel oncolytic adenovirus AdV-D24- inducible co-stimulator ligand (ICOSL)-CD40L was designed and produced to selectively replicate in cancer but not in healthy cells. Indeed, the virus antitumor properties will result in the eradication of cancer cells and, additionally, the production of exogenous proteins that modulate anti-cancer activity through distinct mechanisms that can synergize with other anticancer agents. The OV has been armed with two potent co-stimulatory molecules: inducible co-stimulator ligand (ICOSL) and CD40 ligand (CD40L, CD154). Inducible co-stimulator (ICOS) is a CD28-related molecule expressed on activated T cells, able to interact with its ICOSL present on APCs such as dendritic cells (DCs), B lymphocytes, and some cancer cells [29,30]. Furthermore, ICOSL expression in solid tumors support the activation of CD8+ cytotoxic T cells, thus inducing anti-tumor immune responses [31,32]. Remarkably, it has been also reported that ICOSL transfected tumor cells showed that the ligand positively supports the tumor regression through the activation of CD8+ cytotoxic T-mediated pathways [29,33]. Indeed, Zamarin et al. cloned a recombinant Newcastle disease virus expressing ICOS ligand (NDV-ICOSL) and showed that the injection of NDV-ICOSL in vivo resulted in improved infiltration of activated T cells in both virus-injected and non-injected tumors, leading to the rejection of both tumors when used in combination with CTLA-4 inhibitor [34]. Additionally, it has been also found that CD40 is expressed on B cells, macrophages and DCs and the interaction of CD40–CD40L leads to the activation of adaptive immune responses, including the development of CD8+ cytotoxic T lymphocytes (CTLs) [35]. Moreover, previous studies by Diaconu et al. reported that oncolytic adenovirus coding for CD40L significantly inhibited tumor growth in vivo by exhibiting both oncolytic and apoptotic effects, thus resulting in enhanced calreticulin exposure and high-mobility group box 1 (HMGB1) and ATP release [36]. Taking together these considerations, the presence of both ICOSL and CD40L, which are expressed locally in tumor microenvironment by the vector, should boost the anti-tumor immune responses.

Herein, by exploring the antitumor activity of a novel oncolytic adenovirus AdV-D24-ICOSL-CD40L in combination with anti PD-1 in in vitro and in vivo in immunocompetent mouse model engrafted with murine melanoma cells, we demonstrated that a therapy with OVs expressing potent immune modulators can be an effective strategy to drive the systemic efficacy of an immune checkpoint blockade. Our in vitro and in vivo preclinical findings support the notation that such combination therapy may enhance anti-cancer efficacy and survival via targeted cancer cell lysis and the induction of immunogenic cell death. The outcome of this study may provide important insights to further study the combination therapy approaches using oncolytic adenoviruses and ICIs in solid tumors. Clearly, future investigations will assess the role of ICOSL and CD40L in tumor cells and support possible therapeutic recommendations for the management and treatment of melanoma patients.

## 2. Materials and Methods

### 2.1. Cell Lines, Anti-PD1 Antibodies

Human melanoma cell lines, MUG Mel-1, derived from human brain metastasis, MUG Mel-2 derived from a skin lesion and B16V mouse melanoma cells were kindly provided by Prof. Rinner from the Medical University of Graz and were cultured in RPMI 1640 media (Gibco Laboratories, CA, USA) supplemented with 1% of penicillin/streptomycin (Gibco Laboratories) and 1% L-glutamine (Gibco Laboratories) and 10% fetal bovine serum (FBS, Gibco Laboratories, USA). A549 cells were ordered from the American Type Culture Collection (ATCC, USA) and cultured at 37 °C, 5% CO_2_ in DMEM (Lonza, Switzerland) supplemented with 10% FBS (Gibco Laboratories, USA), 1% of 100 U/mL penicillin/streptomycin (Gibco Laboratories) and 1% L-glutamine (Gibco Laboratories). Pembrolizumab (Keytruda, MSD) and purified anti-mouse CD279 (PD-1) antibody have been resuspended according to manufacturers’ instructions (BioLegend).

### 2.2. Generation and Production of Oncolytic Adenoviruses

The oncolytic virus used in this work was the adenovirus AdV-D24-ICOSL-CD40L, a chimeric serotype 5/3 adenovirus, with a 24-bp deletion in E1A gene and a chimeric fiber knob domain (hybrid 5/3 serotype) and AdV-D24, a vector without co-stimulatory molecules and owning a similar backbone.

The vector AdV-D24-ICOSL-CD40L was designed by authors and purchased (AdenoQuick Kit, OD260 Inc., Boise, OH, USA). AdV-D24 was generated and amplified using adenovirus preparation techniques [37]. The shuttle plasmid containing genes of interest (D24, fiber modifications 5/3, ICOSL + CD40L) were constructed. Subsequently, the cosmid was engineered by the digestion of the shuttle plasmids with SfiI and ligating them to each other. The ligation products were packed into phage lambda, infected *Escherichia coli*, and selected of ampicillin and kanamycin-resistant clones. The identity of obtained colonies was confirmed by restriction analysis or sequencing. Finally, DNA transfection was made in order to rescue the virus by the linearization of the cosmid with PacI or SwaI enzymes. The linear cosmid was transfected into helper cells (human lung cancer cells: A549) and plaques harvested. The virus DNA sequence/genome integrity was confirmed by restriction analysis or sequencing.

Oncolytic adenovirus was characterized by performing titration (VP/mL), expanded in A549 cells and purified on cesium chloride gradients. The OD-260-SDS method was used to determine the concentration of virus particles in the purified stock of viruses (VP/mL). The concentration of virus particles (VPs) in the preparations was calculated given the extinction coefficient of 1.1 × 10^12^ virus particles (VPs) per Abs260 unit in the presence of SDS. The OD-260-reading is a simple, accurate, and precise method to determine adenovirus particle concentration using OD260 nm absorbance. The method guarantees the complete disruption of virus particles and viral DNA prior to absorbance measurements, therefore eliminating absorbance measurement errors. The application of this modified method should reduce interlaboratory variability in determining adenovirus particle concentrations [38]. This method is commonly and widely used in pre-clinical and clinical studies for a dose determination worldwide. The TCID50 assay was also performed to quantify viral titers by determining the concentration at which 50% of the infected cells display cytopathic effect (CPE, IU/mL)). Nevertheless, due to the high variability of this assay, it was not used in downstream analyses. The ratio between VP/IU was also calculated (VP/mL/IU/mL). and ranged from 11 to 13 and 21 (mean 1:17), and was comparable between tested stocks (ratio VP/mL/IU/mL).

### 2.3. Restriction Enzyme Assay (REA)

Viral DNA was extracted from Ad5-D24-ICOSL-CD40L infected A549 cells according to the Hirt method [39,40]. The identity of the virus was assessed by restriction digestion with BamHI and NdeI. All restriction patterns of AdV-D24-ICOSL-CD40L #1-2-3-4 viral DNA match that of the PacI-digested cosmid pAdV-D24-ICOSL-CD40L, indicating the stability of the vector. The presence of the ICOSL-CD40L cassette in the vector was confirmed by the presence of the restriction fragments, highlighted in green in the pictures below.

### 2.4. Whole Genome Sequencing

Viral DNA was extracted from CsCl-purified virus particles using proteinase K. AdV-D24-ICOSL-CD40L sequencing library was made using a Nextera XT kit (Illumina). The size of the library was assessed by high-resolution agarose gel electrophoresis. In order to assess the consensus sequence, the AdV-D24-ICOSL-CD40L library was sequenced on an iSEQ100 instrument (Illumina). The >4.7 m reads (FastQ files) were assembled into a single contig using the program BWA Aligner and the predicted sequence of the AdV-D24-ICOSL-CD40L genome as reference. The contig was visualized using the software Geneious v11.1.5 (Biomatters Ltd., Auckland, New Zealand). The contig calculated a 36,478 bp-long consensus sequence, with a sequence coverage ranging between 911 and ~31,000. In order to derive the sequence alignment, the program SnapGene v5.0.7 was used to align the consensus sequence to the predicted sequence of the virus genome. The alignment confirmed the integrity of the entire genome of AdV-D24-ICOSL-CD40L, including the 24-bp deletion in the E1A CR2 domain, the CMV-ICOSL-IRES-CD40L expression cassette inserted in place of the E3 region and the hybrid Ad5/3 fiber. The presence of the 24-bp deletion in the E1A CR2 domain, the CMV-ICOSL-IRES-CD40L expression cassette inserted in place of the E3 region and the hybrid Ad5/3 fiber were confirmed. Analysis of sequence coverage did not detect the presence of a virus sub-population with a genome characterized by a major rearrangement such as a large insertion or deletion.

### 2.5. Western Blot

The expression of ICOSL and CD40L from AdV-D24-ICOSL-CD40L was assessed by infecting A549 cells with the virus (A549 cells were seeded in 6-cm dishes and were infected with 0.5 mL of crude lysate of viral clones #1-2-3-4) and harvesting proteins 48 h after the infection and detecting ICOSL and CD40L by Western blot. Infected cell pellets were washed twice with cold PBS, then lysed by the addition of ice-cold NP40 cell lysis buffer (Invitrogen #FNN0021). After 30 min incubation at 4 °C with gentle shaking, the cell lysates were clarified by centrifugation (4000 rpm). The protein concentration was measured using a BCA assay (Pierce). CD40L was detected using the primary antibody: goat polyclonal anti-CD40L antibody (R&D #AF617), followed by the secondary antibody: rabbit anti-goat IgG (HRP-conjugated, R&D HAF017). In turn, the ICOSL was detected by staining with primary antibody: mouse monoclonal anti-ICOS ligand antibody (Novus Biologicals # NPB2-46011) and subsequently with the secondary antibody: donkey anti-mouse IgG (HRP-conjugated, R&D HAF018). HRP detection was performed with a SuperSignal West Pico Chemiluminescent substrate (Pierce #34080) and visualized with a Kodak 440CF imager.

### 2.6. CAR and DSG2 Expression in Melanoma Cell Lines

MUG Mel-1 and MUG Mel-2 cells were seeded at the concentration of 5 × 10^5^ cell/well and maintained under the above standard cell culture growth conditions. On the next day, melanoma cells were stained firstly with mouse monoclonal anti-CAR antibody (Santa Cruz Biotech, Dallas, TX, USA) and then with 1:2000 Alexa-Fluor 488 secondary antibody (Abcam, Cambridge, UK) or mouse monoclonal anti-DSG2 antibody (Abcam, Cambridge, UK) and then with 1:2000 Alexa-Fluor 488 secondary (Beckman-Coulter Cytomics FC500).

### 2.7. Cell Viability: MTS Cytotoxicity Assay

Human melanoma cells MUG Mel-1, MUG Mel-2 cells and murine melanoma B16V were seeded at a density of 1 × 10^4^ cells/well in a 96-well plate and maintained under standard growth condition. After overnight incubation, cells were treated as follows: (i) AdV-D24 (0.1, 1, 10, 100 VP/cell), (ii) AdV-D24-ICOSL-CD40L (0.1, 1, 10, 100 VP/cell), (iii) anti PD-1 (100 µg/mL), (iv) AdV5-D24 (100 VP/cell) combined with anti PD-1 (100 µg/mL), (v) Ad5V-D24-ICOSL-CD40L (100 VP/cell) combined with anti PD-1 (100 µg/mL). Cell viability was determined 96 h after treatment, by using CellTiter 96 AQueous One Solution Cell Proliferation Assay (MTS) according to the manufacturer’s instructions (Promega, Madison, WI, USA). The absorbance was measured with a 96-well plate spectrophotometer (Victor Nivo^TM^, PerkinElmer, Milano, Italy) at 490 nm. The experiments were independently performed three times and each treatment was performed in triplicate.

### 2.8. Immunogenicity of Tumor Cell Death In Vitro and Ex Vivo

*Calreticulin (CRT) exposure:* Human and murine melanoma cell lines were seeded in triplicate onto 24-well plates at a concentration of 5 × 10^4^ cells/well and maintained under standard growth condition. On the following day, cells were treated as follows: (i) AdV-D24 (100 VP/cell), (ii) AdV-D24-ICOSL-CD40L (100 VP/cell), (iii) anti PD-1 (100 µg/mL), (iv) AdV5-D24 (100 VP/cell) combined with anti PD-1 (100 µg/mL), (v) Ad5V-D24-ICOSL-CD40L (100 VP/cell) combined with anti PD-1 (100 µg/mL). Then, 48 h after treatment, cells were harvested and stained with 1:1000 diluted Alexa-Fluor 488 rabbit polyclonal anti-calreticulin antibody (Abcam, Cambridge, UK) (concentration of 1 µg/mL) or Alexa Fluor Plus 488 goat anti-mouse at the concentration 1–10 µg/mL (ThermoFisher, Scientific, A32723, Waltham, MA, USA) for 30 min and analyzed by flow cytometry analysis using Beckman-Coulter Cytomics FC500. The experiments were independently performed three times and each treatment was performed in replicates. Additionally, resected tumor tissue from all mice groups (described under the Section 2.10) were collected and processed at sacrifice. A uniform single-cell suspension from tissues were obtained using cell strainers (Corning, 100µm). Subsequently, the single cell suspension was used for the detection of CRT exposure on the cancer cells surface. Cells were harvested and stained by following the same staining protocol as described for the in vitro part above.

*ATP release:* Human and murine melanoma cells were seeded at a concentration of 1 × 10^4^ cells/well in 96-well plates. On the following day, cells were treated as follows: (i) AdV-D24 (100 VP/cell), (ii) AdV-D24-ICOSL-CD40L (100 VP/cell), (iii) anti PD-1 (100 µg/mL), (iv) AdV5-D24 (100 VP/cell) combined with anti PD-1 (100 µg/mL), (v) Ad5V-D24-ICOSL-CD40L (100 VP/cell) combined with anti PD-1 (100 µg/mL). Supernatants were collected after 72 h and analyzed with an ATP detection kit (CellTiter-Glo^®^ Luminescent Cell Viability Assay, Promega) according to the manufacturer’s protocol for luminometric analysis (Victor Nivo^TM^). The experiments were independently performed three times and each treatment was performed in replicates. Additionally, resected tumor tissue from all mice groups (described under the Section 2.10) were collected and processed at sacrifice. A cell suspension from tissues were obtained using cell strainers (Corning, 100 µm). Subsequently, the cell supernatant was used for the detection of ATP release from the cancer cells. Supernatants were collected and analyzed with an ATP detection kit as described for the in vitro part above.

*HGMB-1 release:* Human melanoma cells were seeded at density of 1 × 10^4^ cells per well in 96-well plate and maintained under standard growth condition. On the following day, cells were treated as follows: (i) AdV-D24 (100 VP/cell), (ii) AdV-D24-ICOSL-CD40L (100 VP/cell), (iii) anti PD-1 (100 µg/mL), (iv) AdV5-D24 (100 VP/cell) combined with anti PD-1 (100 µg/mL), (v) Ad5V-D24-ICOSL-CD40L (100 VP/cell) combined with anti PD-1 (100 µg/mL). Supernatants were collected after 72 h and HGMB-1 levels were detected with an Elisa assay kit (MBL International, Woburn, MA, USA), following manufacturer’s instruction.

### 2.9. Evaluation of the Concentration of the ICOSL and CD40L Produced by the Virus

MUG Mel-1 and MUG Mel-2 cells were seeded at 1 × 10^5^ cells/mL in a 96-well plate and maintained under standard growth condition. After overnight incubation, cells were treated as described above. Supernatants were collected 72 h after treatments and analyzed for human ICOSL and CD40L concentration using ELISA kits (LifeSpan BioSciences, Inc, Seattle, WA, USA, LS-F9059, RayBiotech, ELH-CD40L-1, Peachtree Corners, GA, USA) according to the manufacturer’s instructions.

### 2.10. In Vivo Efficacy Studies

All animal procedures were performed and approved by the Austrian Federal Ministry of Science and Research (BMWF) (GZ 66.010/0058-V/3b/2019) and Italian Ministry of Health (117/2020-PR). For the efficacy experiment, murine xenografts were established by subcutaneously (s.c.) injecting 1 × 10^6^ B16V melanoma cells into both flanks of 10-week-old C57BL/6 male mice (6 tumors/group). Tumors (two tumors per mouse, ~5 × 5 mm in diameter) and were randomized prior the treatment initiation as follows: vehicle (100 μL of PBS) administered intratumorally (i.t.), AdV-D24 (administered i.t at a concentration of 1.75 × 10^10^ VP/tumor (3.5 × 10^10^ VP/mouse), AdV-D24-ICOSL-CD40L administered i.t. at a concentration of 1.75 × 10^10^ VP/tumor (3.5 × 10^10^ VP/mouse), murine anti-PD1 (purified anti-mouse CD279 (PD-1) antibody BioLegend) administered intravenously (i.v.) (200 µg/mouse), AdV-D24 (administered i.t. at a concentration of 1.75 × 10^10^ VP/tumor (3.5 × 10^10^ VP/mouse) followed by i.v. treatment with anti-PD1 (200 µg/mouse), AdV-D24-ICOSL-CD40L administered i.t. at a concentration of 1.75 × 10^10^ VP/tumor (3.5 × 10^10^ VP/mouse) followed by i.v. treatment with anti-PD1 (200 µg/mouse) (Table 1). Tumor/lesion size was recorded using caliper on two dimensions every three days. The longest and shortest diameter of tumor at each timepoint were recorded and the tumor volume was calculated using a formula of 0.52 × length 3 (width)^2^. All animals were observed for clinical signs, morbidity or mortality daily during the acclimatization and administration period and additionally 30 min after each treatment.

### 2.11. Statistical Analysis

Data were reported as mean ± SEM or as indicated. Statistical analysis was performed with GraphPad Prism software version 8 (La Jolla, San Diego, CA, USA). An un-paired test and one-way ANOVA with the Krusal–Wallis test was used to compare two or more groups. Survival curves and their statistical analysis were performed using the Kaplan–Meier test.

## 3. Results

### 3.1. Cloning, Characterization, Confirmation of Genetic Stability and Identity of the Double Transgene Vector Expressing Co-Stimulatory Transgenes (ICOSL and CD40L)

Since the treatment with oncolytic viruses is a promising approach for targeting the immune response against the tumor by combining the cytolytic activity together with the ability of the viruses to activate the immune system [41,42], in the current study, we aimed to design and engineer a novel chimeric oncolytic adenovirus vector AdV-D24-ICOSL-CD40L armed with two potent co-stimulatory molecules, ICOSL and CD40L, selectively expressing in cancer cells such as melanoma. The idea behind the insertion of two transgenes is that the targeted delivery of these potent co-stimulatory proteins may improve anti-cancer immune responses to the melanomas and diminish potential undesired off-target effects [32,34,35,36,43,44,45]. To do this, the virus AdV-D24-ICOSL-CD40L replicates in cancer cells whereby the cancer cells are killed via lysis and new virions are released along with the co-stimulatory molecules. In principle, this process can continue as long as there are tumor cells remaining. Previous preclinical data showed that the deletion of 24-bp (D24) of the vector results in a selective replication in cancer cells [37,46]. Specific replication properties of our vectors (AdV-D24-ICOSL-CD40L and AdV-D24) were achieved by the deletion of 24-bp in E1A Conserved Region 2 (CR2). Indeed, the vector was also modified in the fiber knob region, where chimeric oncolytic adenovirus (chimera 5/3, a hybrid Ad5/3 fiber) was engineered and serotype adenovirus 5 fiber knob domain was replaced by serotype knob 3 domain in order to enhance the infectivity in cancer cells [47,48].

Furthermore, the virus was rescued by transfecting PacI-linearized pAdV-D24-ICOSL-CD40L cosmid DNA into A549 cells. Plaques were passaged in A549 cells and their identity was confirmed by the restriction digestion of Hirt DNA with HindIII, BamHI and NdeI (Figure S1). Then, a large-scale amplification/purification of viruses was performed. A total of 1.6 × 10^12^ VP and 2.9 × 10^12^ VP were obtained for two separate batches. The ratio between VP/IU (ratio VP/mL/IU/mL) was calculated and ranged from 11 to 13 or 21 (mean ratio: 1:17) in different stocks. These experiments confirmed that a novel oncolytic adenovirus AdV-D24-ICOSL-CD40L expressing two co-stimulatory transgenes was successfully engineered, purified and produced in a larger scale for further preclinical studies.

#### 3.1.1. Whole Genome Sequencing of the Vector AdV-D24-ICOSL-CD40L

**Figure 1:**
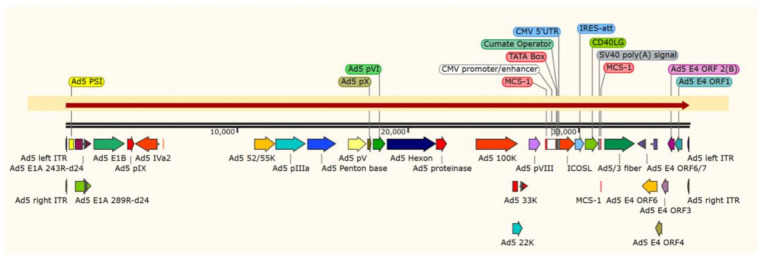
Viral DNA extraction from CsCl-purified virus particles using proteinase K. The AdV-D24-ICOSL-CD40L sequencing library was made using a Nextera XT kit (Illumina). The size of the library was assessed by high-resolution agarose gel electrophoresis. The presence of the 24-bp deletion in the E1A CR2 domain, the CMV-ICOSL-IRES-CD40L expression cassette inserted in place of the E3 region and the hybrid Ad5/3 fiber were confirmed. Analysis of sequence coverage did not detect the presence of a virus sub-population with a genome characterized by a major rearrangement such as a large insertion or deletion. The figure was generated by SnapGene^®^.

The presence of incorporated genetic modification in the genome (D24, fiber 5/3, ICOSL, CD40L) of newly engineered oncolytic vector AdV-D24-ICOSL-CD40L and the genetic stability were assessed by the sequencing of the entire genome of CsCl-purified vector using NGS (Illumina). Whole genome sequencing of the vector AdV-D24-ICOSL-CD40L revealed the presence of the 24-bp deletion in the E1A CR2 domain, the CMV-ICOSL-IRES-CD40L expression cassette inserted in place of the E3 region and the hybrid Ad5/3 fiber (Figure 1). Additionally, the analysis of sequence coverage did not detect the presence of any virus sub-population with a genome characterized by a major rearrangement (large insertion or deletion). The results confirmed the presence of inserted transgenes in the viral genome and the genetic stability.

#### 3.1.2. Expression of CD40L and ICOSL by the Vector AdV-D24-ICOSL-CD40L

**Figure 2:**
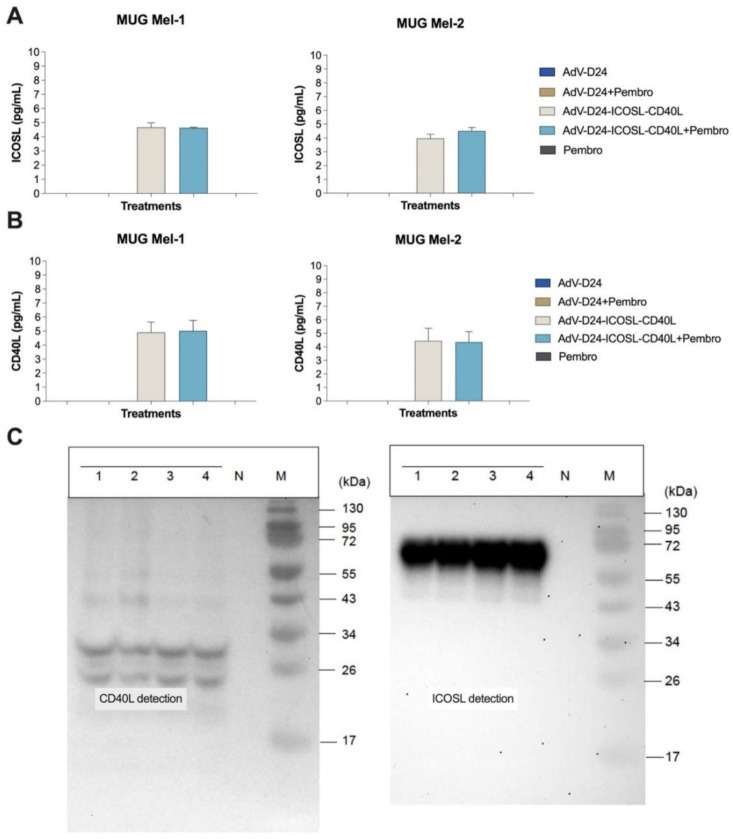
Evaluation of ICOSL and CD40L expression in tested melanoma cells. (**A**) ICOSL concentration was detected on supernatants collected 24–48 h after treatment using ELISA kit. (**B**) CD40L was detected from supernatants collected 24–48 h after treatment using an ELISA kit according to the manufacturer’s instructions. Statistical analysis was carried out with a Mann–Whitney test to compare two groups (ns = not significant, *p* > 0.05). (**C**) Transgene expression. The expression of ICOSL and CD40L from AdV-D24-ICOSL-CD40L was assessed by infecting A549 cells with the virus, harvesting proteins 48 h after the infection and detecting ICOSL and CD40L by Western blot. 1–4: protein extracts from A549 cells infected with AdV-D24-ICOSL-CD40L clones #1–4. N: protein extract from non-infected A549 cells. M: PageRuler Prestained Protein Ladder (ThermoScientific # 26616). ICOSL MW: 70 kDa (due to heavy protein glycosylation); CD40L MW: 30 kDa.

**Figure 3:**
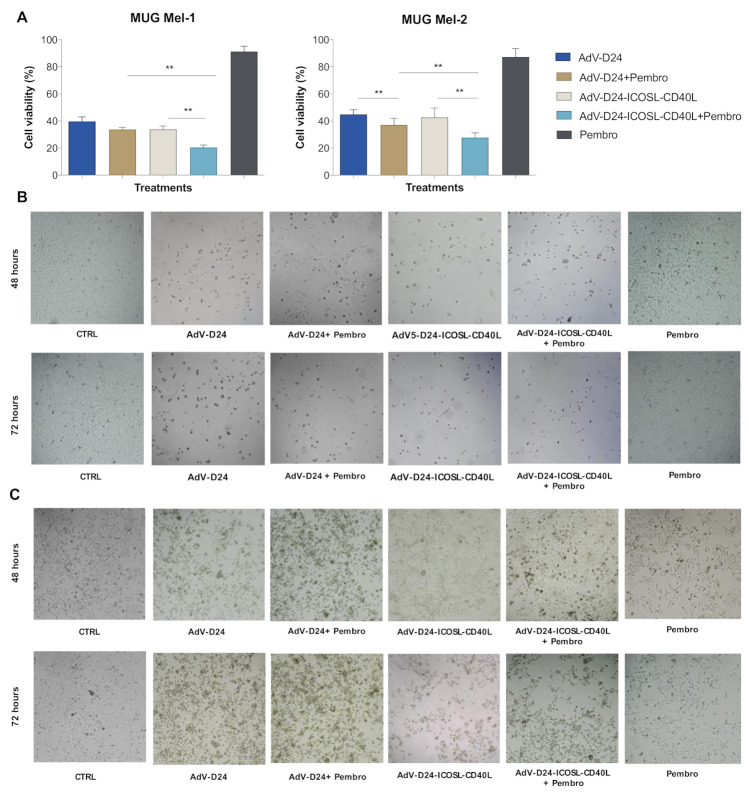
Evaluation of cell viability by MTS assay (cell cytotoxicity assay). (**A**) Cell viability was evaluated 72 h post-infection with AdV-24-ICOSL-CD40L and AdV-24 at the concentration of 100 VP/cell and combination with anti-PD1 (pembrolizumab). Data are expressed as the percentage of viable cells according to MTS cell viability assay protocol (CellTiter 96^®^ AQueous One Solution Cell Proliferation Assay, Promega). Statistical analysis was carried out with a Mann–Whitney test to compare two groups (*p* > 0.05). (**B**,**C**) Microscopic photographs visualizing the morphology and cytopathic effect (CPE) of the cells representing the investigated groups and assessed by MTS assay at 48 and 72 h post-infection (magnification 10×) (** = *p* ≤ 0.001).

Since CD40L and ICOSL exert a role in activation of anti-tumor immune responses via the activation of T cells, we decided to incorporate these two transgenes into the viral backbone. The insertion of both co-stimulatory molecules under the exogenous promoter will allow the virus AdV-D24-ICOSL-CD40L to induce robust and long-lasting immune responses. Therefore, we confirmed, in both human melanoma cell lines MUG Mel-1 and MUG Mel-2, the expressions of CD40L and ICOSL following infection with AdV-D24-ICOSL-CD40L by Western blotting (Figure 2C) and ELISA (Figure 3A,B) analysis after infection in A549 cells.

### 3.2. Expression of Coxsackie-Adenovirus Receptor (CAR) and Desmoglein-2 (DSG-2) Receptors in Human Melanoma Cell Lines MUG Mel-1 and MUG Mel-2

The therapeutic value of oncolytic viruses on melanoma was investigated using MUG Mel-1 and MUG Mel-2 cells by flow cytometry (Beckman-Coulter Cytomics FC500). The expression of adenovirus cell entry specific receptors, namely coxsackie-adenovirus receptor (CAR) and desmoglein-2 (DSG-2), were tested. The results show that the MUG Mel-1 cell line expressed a higher level of DSG-2 receptors (approximately 80% of cells positive for the marker) compared to MUG Mel-2 (approximately 30%). The expression of CAR receptors was much lower in both the cell lines (approx. 4% for both MUG Mel-1 and MUG Mel-2) (Figure S2), thus, suggesting that both metastatic melanoma (Mug Mel-1) and melanoma from the skin (MUG Mel-2) can be targeted with oncolytic adenoviruses exhibiting affinity to DSG-2 receptors [49] for therapeutic treatment.

### 3.3. Evaluation of Cell Viability by MTS Assay (Cell Cytotoxicity Assay)

The infectivity and the cell killing activity of AdV5/3-D24-ICOSL-CD40L was derived with an MTS cell viability assay on human and murine melanoma cell lines. As the control, the oncolytic adenovirus AdV-D24 which does not present neither the chimeric serotype nor the double transgene insertion was used. Following the infection of MUG Mel-1 and MUG Mel-2 cell lines with AdV-24-ICOSL-CD40L and AdV-D24 at the concentration of 100VP/cell, alone and/or in combination with anti PD-1 (pembrolizumab) or only in the presence of pembrolizumab, a reduction in cell viability (expressed in the percentage (%) of viable cells) was observed. The obtained data indicate that the combination therapy of the double transgene vector AdV-24-ICOSL-CD40L with anti PD-1 was the most effective treatment in both cell lines (cell viability was 20.5% and 28% for MUG Mel-1 and MUG Mel-2, respectively). In contrast, the monotherapy with the pembrolizumab was the least effective (91.3% and 87% for MUG Mel-1 and MUG Mel-2, respectively) compared to other tested regimes. Indeed, the combination of AdV-24 and anti PD-1 also showed enhanced cell killing activity over the virus itself: 33.7% (MUG Mel-1) and 37.14% (MUG Mel-2) (Figure 3A). These in vitro results demonstrated that both vectors administered together with pembrolizumab exhibit higher anti-cancer activity with respect to monotherapies. Nevertheless, the efficacy of the combination therapy was higher for the double transgene vector, suggesting higher anti-cancer potency due the presence of the co-stimulatory transgenes. Interestingly, the same trend has been also observed in B16V murine cells, where usually human oncolytic adenoviruses do not optimally replicate, hence the anti-tumor efficacy of AdV-D24 (42.7%), pembrolizumab (94.7%), and AdV-D24-ICOSL-CD40L (41%) individually or in different combinations on B16V cell line was lower than in human melanoma cells (40.4% and 37.3% for AdV-24 plus anti PD-1 and AdV-D24-ICOSL-CD40L plus anti PD-1, respectively) (Figure S3). Notably, both human and murine cells treated only with pembrolizumab showed the least reduction in cell viability (Figure 3A) suggesting that the antibody alone does not have an impact on the cancer cell growth. In line with that, microscopic photographs at 48 and 72 h post-infection (magnification 10×), show changes in the morphology of cells, confirming the anticancer and cytopathic effect (CPE) on cells treated with the different groups (infected with AdV-D24 or AdV-D24-ICOSL-CD40L only or with pembrolizumab or only treated with pembrolizumab) (Figure 3B,C), respectively), thus suggesting that AdV5/3-D24-ICOSL-CD40L alone and or in combination with pembrolizumab could exert an antitumor effect against melanoma.

### 3.4. Immunogenic Cell Death Assessment

**Figure 4:**
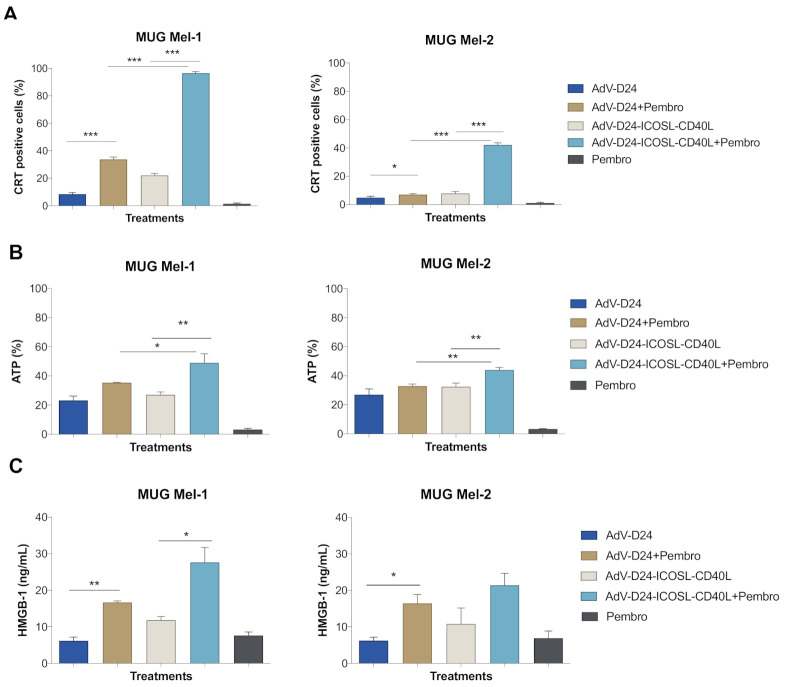
Immunogenic cell death assessment. (**A**) Evaluation of calreticulin (CRT) exposure by melanoma cell lines after treatment with oncolytic adenoviruses AdV-24-ICOSL-CD40L and AdV-D24, and in combination with anti PD-1. CRT exposure was measured 48 h post-treatments with anti-calreticulin antibody staining AlexaFluor^®^ 488 and subsequent flow cytometry analysis (Beckman-Coulter Cytomics FC500). (**B**) Assessment of ATP release after the treatment. ATP concentration in a supernatant was evaluated 72 h after infection with CellTiter-Glo^®^ Luminescent Cell Viability Assay ATP detection kit by Promega. (**C**) Evaluation of high-mobility group box 1 (HMGB-1) release after treatment with oncolytic adenoviruses and the combination with anti PD-1. HMGB-1 level was measured from the supernatant collected 72 h after infection with ELISA kit (MBL International, Woburn, MA, USA), according to manufacturer’s dispositions. Statistical analysis was carried out with a Mann–Whitney test to compare two groups (* = *p* ≤ 0.05; ** = *p* ≤ 0.001, *** = *p* ≤ 0.0001).

To evaluate whether the treatments can trigger immunogenic cell death, the appearance of specific markers, such as the exposure of calreticulin on cell surface and the extracellular release of ATP and HMGB1 [50,51,52], was measured on human melanoma cells treated with (i) AdV-D24 (100 VP/cell), (ii) AdV-D24-ICOSL-CD40L (100 VP/cell), (iii) anti PD-1 (100 µg/mL), (iv) AdV5-D24 (100 VP/cell) combined with anti PD-1 (100 µg/mL), (v) Ad5V-D24-ICOSL-CD40L (100 VP/cell) combined with anti PD-1 (100 µg/mL). The highest immunogenic cell death was observed in the cells infected with AdV-D24-ICOSL-CD40L and treated with pembrolizumab compared to other tested groups (*p* < 0.001, *p* < 0.05, *p* < 0.05, respectively CRT, ATP and HMGB-1 for MUG Mel-1: AdV-D24-ICOSL-CD40L plus pembrolizumab vs. AdV-D24-ICOSL-CD40L; *p* < 0.0001, *p* < 0.001 for, respectively CRT, ATP for MUG Mel-2: AdV-D24-ICOSL-CD40L plus pembrolizumab vs. AdV-D24-ICOSL-CD40L) (Figure 4), while the treatment of cells with only pembrolizumab seemed to not influence the immunogenic cell death program. Nevertheless, it should be also noted that immunogenic cell death markers were significantly higher in cells infected with AdV-D24 and treated with pembrolizumab compared to cells infected only with AdV-D24 in both the cell lines MUG Mel-1 and MUG Mel-2 (*p* < 0.001 for CRT for MUG Mel-1: AdV-D24 plus pembrolizumab vs. AdV-D24; *p* < 0.05, *p* < 0.05 for, respectively, CRT and HMGB-1 for MUG Mel-2: AdV-D24 plus pembrolizumab vs. AdV-D24) (Figure 4). In line with these observations on the immunogenic cell death (ICD) studies, the results on B16V showed a similar trend for the studied markers: ATP and calreticulin (Figure S1).

Moreover, since this study is limited to the tumor cell response in vitro without the contribution of immune cells that play a crucial role in the development and maintenance of ICD, we concluded that the virus combined with pembrolizumab were able to block the growth of tumor cells and induce immunogenic cell death according to another mechanism different than the immune cell-mediated one. Finally, these findings highlight that the extent of immunogenic cell death was more remarkable (*p* ≤ 0.05) for the combination of the double transgene vector with anti PD-1, suggesting their immunomodulatory properties in vitro.

### 3.5. Antitumor Efficacy of AdV-D24-ICOSL-CD40L and the Combination Therapy with Anti PD-1 Antibody in Murine Melanoma B16V Allograft Immunocompetent C57BL/6 Model

The ability of the combination treatment using oncolytic viruses with pembrolizumab to enhance anti-cancer potency and induce immunogenic cell death were investigated in vivo. To this aim, we used the C57BL/6 as the immunocompetent mouse model rather than immunodeficient xenograft alternative models [53] since we aimed to elucidate the involvement and contribution of the immune system to the efficacy of our combination therapy. Syngeneic tumor models (C57BL/6 or BALB/c mice) are the oldest and most commonly utilized preclinical models to evaluate anticancer therapeutics. The fully immunocompetent models are useful in the assessment of immuno-oncology agents (ICIs, oncolytic vectors) in order to study the development of antitumor immune responses, plus they do not require the adoptive transfer of immune cells [54,55].

Animal studies in syngeneic melanoma mouse model were performed in order to evaluate the possible antitumor effects triggered by the different treatments planned, according to the schedule presented in Table 1.

**Figure 5:**
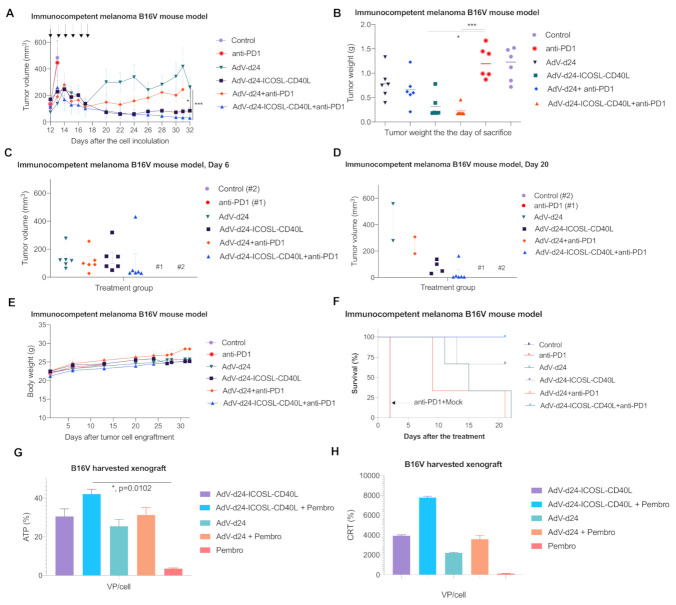
Antitumor efficacy of oncolytic vectors and the combination therapy with anti PD-1 antibody in murine melanoma B16V (1 × 10^6^ cells/flank, one mouse had 2 tumors/6 tumors per group) xenograft immunocompetent C57BL/6 model. (**A**) Tumor volume (mm^3^) measured through the study. The treatment was performed once per day on days 1–6. The mice were treated according to the scheme (Table 1) with viruses (i.t.) and anti PD-1 antibody (i.v.) (**B**) At the end of the study, mice were sacrificed and tumors harvested for weight assessment. (**C**,**D**) Tumor volume measurement on days 6 and 20. (**E**) Body weight measurements throughout the study. (**F**) Survival profile was calculated by Kaplan–Meier test. (**H**) Evaluation of CRT exposure after the treatment with oncolytic adenoviruses AdV-24-ICOSL-CD40L and AdV-D24, and in combination with anti PD-1. CRT exposure was measured in the end of the study (after mice sacrifice) with anti-calreticulin antibody staining and subsequent flow cytometry analysis (Beckman-Coulter Cytomics FC500). (**G**) Assessment of ATP release after the treatment. ATP concentration from the tumors was evaluated in the end of the study (after mice sacrifice) with CellTiter-Glo^®^ Luminescent Cell Viability Assay ATP detection kit by Promega. Error bars, mean ± SEM, * = *p* ≤ 0.05, *** = *p* ≤ 0.0001.

Interestingly, the highest decrease in tumor volume growth rate was detected in the group of mice treated with AdV-D24-ICOSL-CD40L in combination with anti PD-1 (31.3 mm^3^) with a significant difference when compared to the group treated with AdV-D24 plus anti PD-1 (244 mm^3^) (*p* < 0.001 for tumor volume) and the group treated with anti PD-1 (445 mm^3^) (Figure 5A,B). Nevertheless, also the mice treated only with the virus AdV-D24-ICOSL-CD40L resulted in significant tumor volume eradication (85 mm^3^, *p* < 0.05) compared to the group with anti-PD-1. Additionally, the vector AdV-D24 alone or in combination with anti PD-1 antibody also showed anticancer efficacy in comparison to anti-PD1 antibody alone, where no effect was detected thus suggesting that the B16V melanoma seems to be refractory to the ICI therapy (261; 244; 445 mm^3^, respectively for AdV-D24, AdV-D24 plus anti PD-1 and PD-1 alone). However, the most effective regimen in terms of tumor volume was the treatment with AdV-D24-ICOSL-CD40L in combination with anti PD-1 antibody on day 6 and day 21 (Figure 5C,D) thus indicating the most significant antitumor activity compared to other groups.

Notably, all the mice well tolerated the treatment and no signs of toxicity and weight loss were detected during the treatment course, thus justifying the benefits in survival profile, especially for the group of mice treated with viruses and anti-PD1 antibody. Remarkably, the group injected with AdV-D24-ICOSL-CD40L and treated in combination with anti PD-1 antibody resulted in 100% survival (Figure 5E,F), thus suggesting it is the most efficacious therapy with a safe profile.

It has been shown that cancer cell death can be immunogenic or non-immunogenic. Immunogenic cell death (ICD) comprises changes in the structure of the cell surface and leads to the release of proimmunogenic factors. Subsequently, it attracts APCs to take up tumor antigens, process them and finally elicit an anti-tumor immune response (specific anti-tumor T cells). ICD can be evaluated by the presence of ICD biomarkers such as calreticulin (CRT) in the outer plasma membrane, followed by the extracellular release of high-mobility group box 1 protein (HMGB1) and adenosine triphosphate (ATP). Therefore, in our studies we have assessed the expression of the ICD markers in order to study the immunogenicity caused by the treatment. In fact, the in vitro model did not mimic optimal conditions due to the lack of immune system, which is crucial in the development of ICD [56]. Therefore, we conduced ex vivo analyses towards further assessment of the ICD based on the tumor tissue extracted from the treated mice. Our ex vivo results showed that the combinatory therapy (oncolytic adenovirus plus anti PD-1) resulted in the most profound expression of the ICD markers (ATP: AdV-D24-ICOSL-CD40L + pembro vs. pembro, *p* < 0.05) (Figure 5G,H).

Our findings indicate that the combination therapy might boost anti-cancer immune responses and highlight the potential of the oncolytic vectors as an immunosensitizing agent for combination therapies with checkpoint inhibitors, as also reported by Ranki et al. [57]. Importantly, this effect can be further enhanced by arming oncolytic vectors with properly selected co-stimulatory molecules such as CD40L and ICOSL.

## 4. Discussion

The low 5-year survival rate in metastatic melanoma (around 20%) [58] suggests that the use of immune checkpoint inhibitors (ICIs) such as pembrolizumab has provided benefits only in a small number of cases [4,59]. Indeed, for the majority of the patients, newer treatment options, either alone or in combination with already approved treatment modalities, are urgently required [60]. Although ICIs have enhanced the survival of some melanoma cancer patients by a few years, there are still many cases resistant to ICI treatments [61]. Furthermore, about 25% of the responders, despite ongoing therapy, develop relapses due to treatment resistance [25,62]. Nevertheless, metastatic melanoma responds better to immunotherapy compared to conventional chemotherapeutic drugs and radiotherapy [63,64,65]. Hence, combined treatment with novel oncolytic adenovirus and ICIs induces complementary antitumor response that could actually lead to more favorable therapeutic outcomes [66,67,68,69]. Indeed, pre-clinical and clinical findings show that oncolytic vectors can trigger anti-tumor immunity and increase immune cell infiltration (including cytotoxic CD8+ T cells) into the tumor microenvironment (TME). Priming with oncolytic vector can switch a “cold” TME into a “hot” one in terms of the infiltration of various innate and adaptive immune cell subsets into a tumor lesion. Therefore, this observation sets the stage for combination therapy with ICIs as they are most effective in a TME with a presence of lymphocytic infiltrates [7]. Indeed, it is conceivable that oncolytic vectors plus ICIs will be used as a combination therapy for multiple types of cancers, including melanoma, thus a considerable spectrum of various genes has already been integrated in oncolytic adenoviruses to enhance immune stimulation including antigen presentation, T cell priming or counterplay with immunosuppression. Such critical concepts have the potential to play a promising future role as enablers of immunotherapies involving oncolytic vectors [70,71].

Although extensive studies have been conducted to improve melanoma therapy [72,73], the treatment of advanced melanoma malignancies still presents a few challenges such as the need to refine the efficacy, safety and tolerability of ICIs. Oncolytic viruses (OVs) have demonstrated their ability to provide a synergistic anticancer effect in combination with ICIs, chemotherapy and radiotherapy [15,50,71,74,75,76]. Although OVs show clinical promise in already immunogenic malignancies and a safe profile, clinical response rates are inconsistent [77]. Therefore, the efficacy of oncolytic vectors needs to be strengthened in order to translate them as a mainstream in cancer therapy. One of the strategies to enhance the therapeutic efficacy of OVs is their use as primers for other immunotherapies. To this point, genes encoding for immunomodulatory proteins are the most commonly studied for arming oncolytic vectors and have the potential to widen the applications of immuno-oncology (IO) in cancers such as melanoma [69]. Therefore, we designed a strategy to engineer a newly oncolytic vector AdV-D24-ICOSL-CD40L and investigated its anti-tumor activity in combination with anti PD-1 in in vitro and in vivo immunocompetent mouse model engrafted with murine B16V melanoma cells. Moreover, the novel AdV-D24-ICOSL-CD40L has been produced to purposefully infect cancer cells through the DSG-2 receptor. Indeed, in order to target tumor cells, the expression of the adenovirus receptor on the tumor cell surface is crucial [78,79]. Hence, it has been reported that different adenovirus serotypes bind various receptors on the cell of which the expression can vary between tumor types. Moreover, adenovirus 5 infects the cells through the CAR receptor, while adenovirus 3 binds to the DSG-2 and CD46 instead [79]. Accordingly, in our experiments, the expression level of the primary entry receptors has been assessed in both tested melanoma cell lines: MUG Mel-1 and MUG Mel-2. The obtained results demonstrated that the MUG Mel-1 cell line expressed a higher level of DSG-2 receptors (approx. 80% of the cells express DSG-2) compared to MUG Mel-2 (approx. 30%). In turn, the expression of the CAR receptor was low in both the cell lines (4.4% and 4.0%, respectively for MUG Mel-1 and MUG Mel-2), thus supporting the rationale for the treatment of melanoma using oncolytic vector AdV-D24-ICOSL-CD40L [49].

Moreover, cell viability assays, carried out with both human melanoma cell lines, revealed that combination therapy using AdV-D24-ICOSL-CD40L with pembrolizumab led to higher anti-cancer activity compared to the other tested groups. Interestingly, cell viability was affected the least in all of the in vitro models treated only with anti PD-1 antibody. This is in agreement with our previous studies showing that the addition of pembrolizumab along with oncolytic adenovirus AdV5/3-D24-GM-CSF in SK-Mel-28 cells led to a stronger anti-tumor effect in comparison to single therapies [80].

It is well known that oncolytic adenoviruses are able to induce immunogenic cancer cell death (ICD) and the release of tumor specific antigens for APC cells, triggering the priming of potent tumor-specific immunity [70]. Hence, the combination of oncolytic vectors with other therapeutic agents has the potential for enhanced anticancer efficacy and the development of antineoplastic immunity, thus representing a powerful tool to overcome the major obstacle of an immune suppressive microenvironment and the subsequent induction of anti-cancer immune responses [81]. While assessing the extent of ICD, it has been found that the level of calreticulin exposure (CRT positive cells in %), and ATP (in %) and HMGB-1 (in %) release were significantly elevated in both cell lines treated with AdV-D24-ICOSL-CD40L in combination with pembrolizumab as compared to the other groups, except for the HMGB-1 level in MUG Mel-2 cells where the level was higher, but the difference was not significant (*p* < 0.001, *p* < 0.05, *p* < 0.05 for, respectively CRT, ATP and HMGB-1 for MUG Mel-1: AdV-D24-ICOSL-CD40L plus pembrolizumab vs. AdV-D24-ICOSL-CD40L; *p* < 0.0001, *p* < 0.001 for, respectively CRT, ATP for MUG Mel-2: AdV-D24-ICOSL-CD40L plus pembrolizumab vs. AdV-D24-ICOSL-CD40L) and this trend was also confirmed in B16V cells (ATP: AdV-D24-ICOSL-CD40L + pembro vs. pembro, *p* < 0.05). Interestingly, in line with our findings, it is reported by the literature that the combination therapy of oncolytic adenoviruses with chemotherapeutic drugs and ICIs resulted in the enhanced expression of calreticulin on the cancer cell surface and a higher release of both ATP and HMGB-1 markers in mesothelioma and melanoma therapies [50,80]. Indeed, it is postulated that ICD constitutes a prominent pathway for the activation of the immune responses against cancer, which in turn affect the long-term success of anti-cancer therapies along with the long-lasting protective anti-tumor immunity [51]. Taken together, these results may suggest an enhanced cytotoxic immune effect induced by AdV-D24-ICOSL-CD40L and pembrolizumab compared to other groups. Certainly, the impact of these effects has to be further investigated in additional mouse models (e.g., humanized) by focusing on immune responses, the infiltration of immune cells to the injected lesions and T cell phenotyping, but this was not the primary aim of this work.

The interplay among cancer cells, immune cells and anticancer drugs is complex. Indeed, the success of immunotherapy has changed the practice of cancer treatment tremendously [82]. However, there are still many challenges, such as drug resistance, the assessment of combinatory therapies, biomarker discovery, the prediction of adverse events in preclinical settings and others. To overcome these challenges, it is crucial to develop reliable preclinical mouse models that recapitulate the clinical features, biological heterogeneity, and immune microenvironment of human cancers [83]. Selecting the right mouse model to study these interactions is a key step for the development of immunotherapies [84]. Therefore, in order to assess the anti-cancer potential and clinical benefits of the combination therapy of oncolytic adenoviruses and ICIs, we have developed an immunocompetent melanoma B16V mouse model.

Evaluation of antitumor efficacy of AdV-D24-ICOSL-CD40L, AdV-D24 and the combination therapy with anti PD-1 antibody in murine melanoma B16V allograft immunocompetent C57BL/6 model revealed maximum and significant reduction in tumor volume in mice treated with AdV-D24-ICOSL-CD40L combined with anti PD-1 antibody. Remarkably, the combination therapy resulted in 100% survival and no loss in body weight. This is a promising result, showing that the investigated combination treatment regimen might extend melanoma cancer patient survival and present a well-tolerated safety profile. In addition, the ex vivo investigation of ICD profile evaluated on explanted murine tumor tissues confirmed that the combinatory therapy resulted in the most profound expression of the ICD markers. Nevertheless, despite these findings, our in vivo study has some limitations as the human oncolytic adenoviruses are not optimally replicating in murine tissue [85,86]. The efficient replication of human adenoviruses in murine cells is significantly lower than in human cells, although infection can be detectable. However, the mechanisms for a low level of infectious virions remain unclear [87]. Therefore, this limitation could have affected the anti-cancer efficacy of the viruses itself in the B16V mouse model. Nevertheless, on the other hand, oncolytic viruses in addition to oncolytic properties exhibit the ability to induce anti-cancer immune responses, which can lead to the eradication of cancer cells. However, the strength of the selected model and the results is the presence of a fully functional immune system in C57BL/6 mice allowing for a proper induction, development and modulation of immune responses against cancer cells and the prevention of the inhibition of effector T cells.

In a similar study, a rational combination approach aimed at exploring the role of immunogenic oncolytic adenovirus Ad5/3-D24-GM-CSF with pembrolizumab in humanized A2058 melanoma huNOG mouse model showed a significant reduction in tumor volume compared to pembrolizumab treatment alone without lowering body weight [80]. Notably, another study by Bramante S. et al. evaluated the effects of 5/3 chimeric oncolytic adenovirus coding for GM-CSF with low dose cyclophosphamide in in vitro and in vivo melanoma models [88] revealing a promising outcome following treatment with the oncolytic virus, thus advocating the need for more studies exploring the use of oncolytic viral therapy able to support the future design of clinical trials and to enhance the efficacy of cancer treatment, including melanoma. For instance, ongoing clinical trials are currently exploring the role of oncolytic virus ONCOS-102 in combination with ICIs in melanoma cancer cases refractory to the treatment (NCT03003676). In this phase 1 trial, the combination of ONCOS-102 and pembrolizumab has been tested in patients with advanced, unresectable melanoma who have had disease progress despite treatment with anti-PD1. It has been reported that 7 out of 20 patients treated with ONCOS-102 and pembrolizumab combination resulted in an ORR of 35% by RECIST 1.1 criteria. Importantly, it was also observed that multiple non-injected lesions completely disappeared, indicating that ONCOS-102 can induce systemic anti-tumor immunity [89]. Interestingly, Frohlich et al. also reported that the oncolytic vector T-VEC showed the potential for synergistic anti-cancer effects to overcome the resistance of mucosal melanoma to ICI anti PD-1 [90]. Other studies also support combining ICIs with oncolytic vectors. Particularly, the combination of ICIs with T-VEC may result in a synergistic anti-cancer efficacy for patients with unresectable melanoma as a higher ORR and complete response rate (CRB) was reported compared to published studies on similar therapeutic regimens [28]. In another clinical study on metastatic melanoma patients, treatment with T-VEC showed a better durable response rate (continuous complete response or partial response lasting ≥ 6 months) over subcutaneous GM-CSF (16.3% vs. 2.1%; *p* < 0.001). Importantly, responses were observed in both injected and untreated lesions. Indeed, when T-VEC was combined with ICIs, significantly improved response rates were reported (compared to monotherapies). Similar findings were observed with combinations of ICIs and other oncolytic vectors such as CAVATAK with ipilimumab [91].

These findings highlight the potential of the oncolytic vectors as an immunosensitizing agent for combination therapies with checkpoint inhibitors in a group of ICI refractory patients [57].

Nevertheless, the promising results obtained from the published studies on the efficacy and safety of oncolytic viruses for the treatment of different types of cancer (ovarian, mesothelioma, melanoma, etc.) underlines the need for further studies aimed at exploring the roles of different oncolytic viruses in both preclinical and clinical settings. To the best of our knowledge, ours is the first study investigating the oncolytic role of AdV-D24-ICOSL-CD40L in melanoma preclinical studies. Even if this represents an open line of investigation, as further studies should be carried out to better elucidate the role of AdV-D24-ICOSL-CD40L in melanoma, it represents a milestone for development. Future studies will leverage the oncolytic and immunomodulatory properties of AdV-D24-ICOSL-CD40L in combination with other anticancer drugs complementing the anti-cancer properties, such as adoptive T cell therapy (ACT) and bispecific antibodies (BsAb), in order to maximize treatment against cancer.

## 5. Conclusions

We have successfully engineered a novel oncolytic adenovirus AdV-D24-ICOSL-CD40L expressing co-stimulatory molecules: ICOSL and CD40L. Our preclinical results strengthen the notion that combination therapy of oncolytic adenovirus with ICIs may enhance anti-cancer efficacy and survival via targeted cancer cell lysis and the induction of immunogenic cell death in melanoma models. These data may provide important insights to further study the combination therapies in solid tumors. Future studies will assess the role of ICOSL and CD40L in tumor cells and support possible recommendations for the management and treatment of melanoma-bearing patients. Notably, we have proposed a new treatment strategy against melanoma to be further investigated.

## Figures and Tables

**Table 1:** Overview of the treatment scheme. i.t: intratumorally; i.v: intravenously; ICOSL: inducible co-stimulator ligand.

Group	Tumor Cell Engraftment	Day 1	Day 2	Day 3	Day 4	Day 5	Day 6
1. Mock	Tumor inoculation: 2 tumors per mouse, one tumor/flank. Each flank was engrafted with 1 × 10^6^ B16V cells in 50–100 µL (6 tumors per group)	PBS i.t. PBS i.v.	PBS i.t. PBS i.v.	PBS i.t. PBS i.v.	PBS i.t. PBS i.v.	PBS i.t. PBS i.v.	PBS i.t. PBS i.v.
2. AdV-D24-ICOS-CD40L		Virus i.t.	Virus i.t.	Virus i.t.	Virus i.t.	Virus i.t.	Virus i.t.
3. AdV-D24-WT		Virus i.t.	Virus i.t.	Virus i.t.	Virus i.t.	Virus i.t.	Virus i.t.
4. Anti PD-1		200 µg i.v.	200 µg i.v.	200 µg i.v.	200 µg i.v.	200 µg i.v.	200 µg i.v.
5. AdV-D24-ICOS-CD40L + anti PD-1		Virus i.t. + 200 µg i.v.	Virus i.t. + 200 µg i.v.	Virus i.t. + 200 µg i.v.	Virus i.t. + 200 µg i.v.	Virus i.t. + 200 µg i.v.	Virus i.t. + 200 µg i.v.
6. AdV-D24-WT + anti PD-1		Virus i.t. + 200 µg i.v.	Virus i.t. + 200 µg i.v.	Virus i.t. + 200 µg i.v.	Virus i.t. + 200 µg i.v.	Virus i.t. + 200 µg i.v.	Virus i.t. + 200 µg i.v.

## Data Availability

Not applicable.

## Supplementary Materials

The following are available online at https://www.mdpi.com/article/10.3390/pharmaceutics13040547/s1, Figure S1. Virus rescue and characterization. Viral DNA was extracted from AdV-D24-ICOSL-CD40L infected A549 cells according to the Hirt method. The identity of the virus was assessed by restriction digestion with HindIII A, BamHI and NdeI B. All restriction patterns of AdV-D24-ICOSL-CD40L #1-2-3-4 vDNA match that of the PacI-digested cosmid pAdV-D24-ICOSL-CD40L, indicating the stability of the vector. The presence of the ICOSL-CD40L cassette in the vector was confirmed by the presence of the restriction fragments highlighted in green in the pictures. A: pAdV-D24-ICOSL-CD40L/PacI, B: pAdV-D24-ICOSL-CD40L, C: pAdV-D24-ICOSL-CD40L/PacI, D: pAdV-D24-ICOSL-CD40L. The identity of the vector was confirmed by restriction digestion of Hirt DNA with HindIII, BamHI and NdeI; Figure S2. Expression of coxsackie-adenovirus receptor (CAR) and desmoglein-2 (DSG-2) receptors in human melanoma cell lines MUG Mel-1 and MUG Mel-2, measured with flow cytometry with Beckman-Coulter Cytomics FC500. Data are expressed as percentage of cell positive for the marker; Figure S3. Evaluation of cell viability by MTS assay (cell cytotoxicity assay) and immunogenic cell death. A Cell viability was evaluated 72 h post-infection with AdV-D24-ICOSL-CD40L and AdV-D24 at the concentration of 100 VP/cell and combination with anti-PD1 in murine B16V melanoma cell line. Data are expressed as percentage of viable cells according to MTS cell viability assay protocol (CellTiter 96^®^ AQueous One Solution Cell Proliferation Assay, Promega). Immunogenic cell death assessment. B Assessment of ATP release after the treatment. ATP concentration in a supernatant was evaluated 72 h after infection with CellTiter-Glo^®^ Luminescent Cell Viability Assay ATP detection kit by Promega. C Evaluation of CRT exposure by melanoma cell lines after treatment with oncolytic adenoviruses AdV-24-ICOSL-CD40L and AdV-D24, and in combination with anti PD-1. CRT exposure was measured 48 h post-treatments with anti-calreticulin staining and subsequent flow cytometry analysis (Beckman-Coulter Cytomics FC500). Statistical analysis was carried out with a Mann–Whitney test to compare two groups (* = *p* ≤ 0.05; ** = *p* ≤ 0.001).
