# Supplementary material for: Combination Therapy of Novel Oncolytic Adenovirus with Anti-PD1 Resulted in Enhanced Anti-Cancer Effect in Syngeneic Immunocompetent Melanoma Mouse Model
Source: Pharmaceutics. 2021 Apr 14;13(4):547. doi: 10.3390/pharmaceutics13040547 (PMC8070801; doi:10.3390/pharmaceutics13040547)
Supplement: Supplementary file 1 [file pharmaceutics-13-00547-s001.pdf]

pharmaceutics

MDPI

Supplementary Materials: Combination Therapy of Novel Oncolytic Adenovirus with Anti-PD1 Resulted in Enhanced Anti-Cancer Effect in Syngeneic Immunocompetent Melanoma Mouse Model

Mariangela Garofalo, Laura Bertinato, Monika Staniszewska, Magdalena Wieczorek, Stefano Salmaso, Silke Schrom, Beate Rinner, Katarzyna Wanda Pancer and Lukasz Kuryk

Citation: Garofalo, M.; Bertinato, L.; Staniszewska, M.; Wieczorek, M.; Salmaso, S.; Schrom, S.; Rinner, B.; Pancer, K.W.; Kuryk, L. Combination Therapy of Novel Oncolytic Adenovirus with Anti-PD1 Resulted in Enhanced Anti-Cancer Effect in Syngeneic Immunocompetent Melanoma Mouse Model. *Pharmaceutics* **2021**, *13*, 547. https://doi.org/10.3390/pharmaceutics13040547

Academic Editor: Christopher Scott

Received: 26 March 2021
Accepted: 11 April 2021
Published: 14 April 2021

Publisher's Note: MDPI stays neutral with regard to jurisdictional claims in published maps and institutional affiliations.

Copyright: © 2021 by the authors. Submitted for possible open access publication under the terms and conditions of the Creative Commons Attribution (CC BY) license (http://creativecommons.org/licenses/by/4.0/).

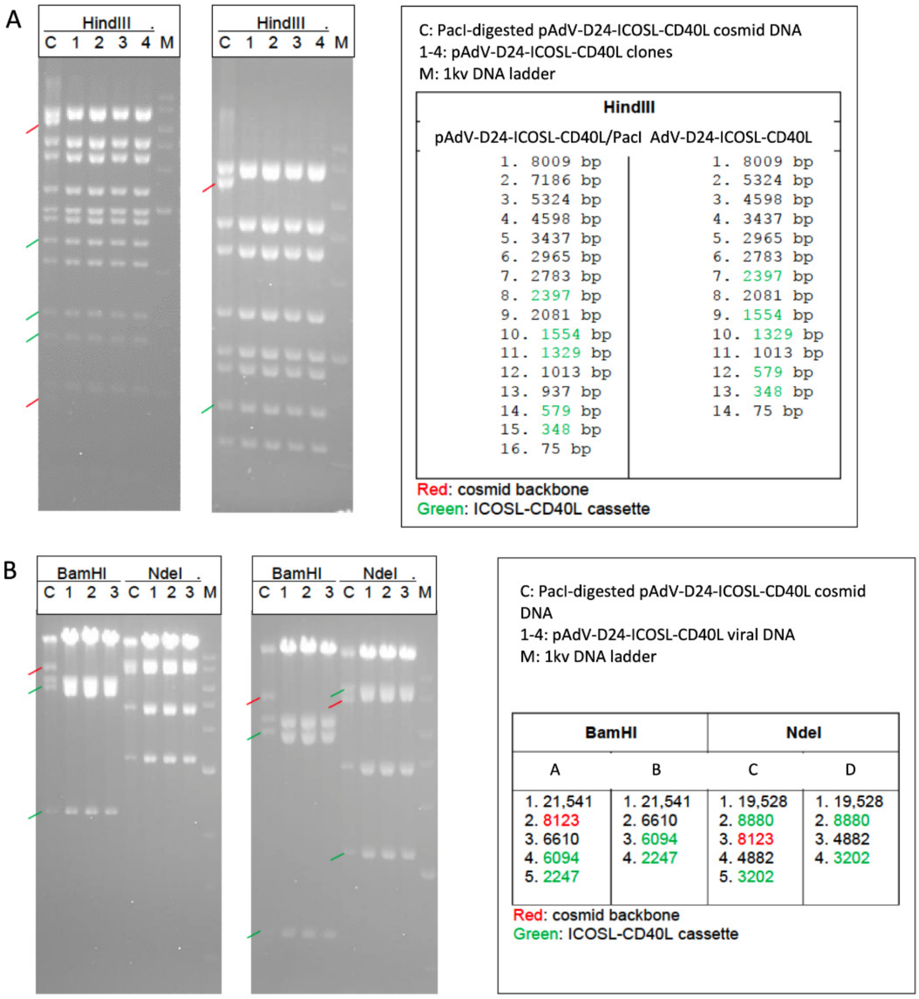

Figure S1. Virus rescue and characterization. Viral DNA was extracted from AdV-D24-ICOSL-CD40L infected A549 cells according to the Hirt method. The identity of the virus was assessed by restriction digestion with HindIII (**A**), BamHI and NdeI (**B**). All restriction patterns of AdV-D24-ICOSL-CD40L #1-2-3-4 vDNA match that of the PacI-digested cosmid pAdV-D24-ICOSL-CD40L, indicating the stability of the vector. The presence of the ICOSL-CD40L cassette in the vector was confirmed by the presence of the restriction fragments highlighted in green in the pictures. A: pAdV-D24-ICOSL-CD40L/PacI, B: pAdV-D24-ICOSL-CD40L, C: pAdV-D24-ICOSL-CD40L/PacI, D: pAdV-D24-ICOSL-CD40L. The identity of the vector was confirmed by restriction digestion of Hirt DNA with HindIII, BamHI and NdeI.

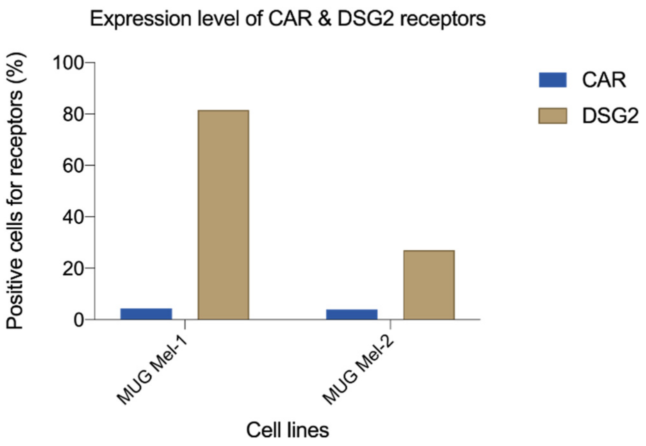

Figure S2. Expression of Coxsackie-Adenovirus Receptor (CAR) and Desmoglein-2 (DSG-2) receptors in human melanoma cell lines MUG Mel-1 and MUG Mel-2, measured with flow cytometry with Beckman-Coulter Cytomics FC500. Data are expressed as percentage of cell positive for the marker.

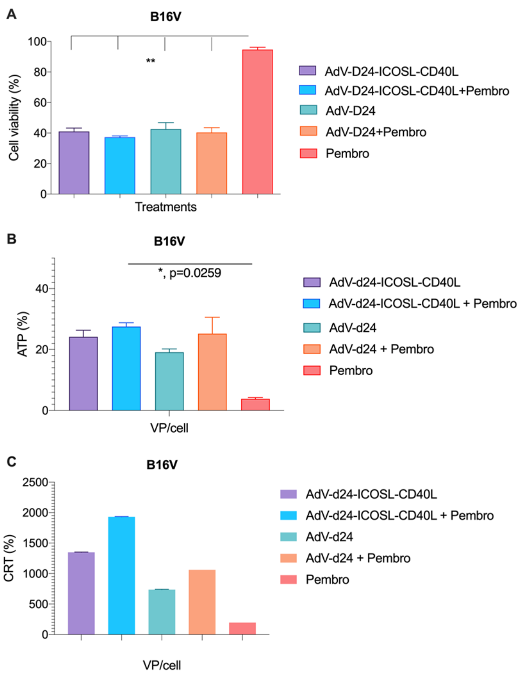

Figure S3. Evaluation of cell viability by MTS assay (cell cytotoxicity assay) and immunogenic cell death. (**A**) Cell viability was evaluated 72 h post infection with AdV-D24-ICOSL-CD40L and AdV-D24 at the concentration of 100VP/cell and combination with anti-PD1 in murine B16V melanoma cell line. Data are expressed as percentage of viable cells according to MTS cell viability assay protocol (CellTiter 96® AQueous One Solution Cell Proliferation Assay, Promega). Immunogenic cell death assessment. (**B**) Assessment of ATP release after the treatment. ATP concentration in a supernatant was evaluated 72 h after infection with CellTiter-Glo® Luminescent Cell Viability Assay ATP detection kit by Promega. (**C**) Evaluation of CRT exposure by melanoma cell lines after treatment with oncolytic adenoviruses AdV-24-ICOSL-CD40L and AdV-D24, and in combination with anti PD-1. CRT exposure was measured 48 h post treatments with anti-calreticulin staining and subsequent flow cytometry analysis (Beckman-Coulter Cytomics FC500). Statistical analysis was carried out with a Mann-Whitney test to compare two groups (* = $p \leq 0.05$; ** = $p \leq 0.001$).
